# A combined system of microbial fuel cell and intermittently aerated biological filter for energy self-sufficient wastewater treatment

**DOI:** 10.1038/srep18070

**Published:** 2015-12-15

**Authors:** Yue Dong, Yujie Feng, Youpeng Qu, Yue Du, Xiangtong Zhou, Jia Liu

**Affiliations:** 1 State Key Laboratory of Urban Water Resource and Environment, Harbin Institute of Technology. No 73 Huanghe Road, Nangang District, Harbin 150090, China; 2 School of Life Science and Technology, Harbin Institute of Technology. No. 2 Yikuang Street, Nangang District, Harbin 150080, China

## Abstract

Energy self-sufficiency is a highly desirable goal of sustainable wastewater treatment. Herein, a combined system of a microbial fuel cell and an intermittently aerated biological filter (MFC-IABF) was designed and operated in an energy self-sufficient manner. The system was fed with synthetic wastewater (COD = 1000 mg L^−1^) in continuous mode for more than 3 months at room temperature (~25 °C). Voltage output was increased to 5 ± 0.4 V using a capacitor-based circuit. The MFC produced electricity to power the pumping and aeration systems in IABF, concomitantly removing COD. The IABF operating under an intermittent aeration mode (aeration rate 1000 ± 80 mL h^−1^) removed the residual nutrients and improved the water quality at HRT = 7.2 h. This two-stage combined system obtained 93.9% SCOD removal and 91.7% TCOD removal (effluent SCOD = 61 mg L^−1^, TCOD = 82.8 mg L^−1^). Energy analysis indicated that the MFC unit produced sufficient energy (0.27 kWh m^−3^) to support the pumping system (0.014 kWh m^−3^) and aeration system (0.22 kWh m^−3^). These results demonstrated that the combined MFC-IABF system could be operated in an energy self-sufficient manner, resulting to high-quality effluent.

The energy crisis and environment pollution are two major challenges facing the world today. Microbial fuel cell (MFC) is an infant but promising technology to help partially address these challenges^1^. It has been widely studied in exoelectrogen^2^, electrode materials^3^, reactor configurations^4^, and so forth. A primary function of MFC technology is wastewater treatment. However, the issues surrounding MFC effluent quality have not yet been sufficiently addressed. MFC alone may not be a feasible avenue to meet stringent effluent quality requirements, hence requiring another step such as integration of MFC with membrane technology^5^ or conventional treatment technology^6^ to further purify the treated effluent. Furthermore, direct electricity generation is an integral characteristic of MFC. Typical MFC systems are known to generate power at milliwatt (mW) level, depending on influent characteristic, reactor configuration, and operating parameter. This low and unstable power output has been a large obstacle in preventing MFC as a renewable power source accessing the power grid which is in kW or MW level of installed capacity in conventional power generation. So, lack of proper accountability of electricity generation has attracted more attention in recent years.

A possible strategy was proposed based on *in-situ* utilization of the generated electricity for energy self-sufficient wastewater treatment process with MFC-based combined system^7^. The potential energy stored within different wastewater is variable, ranging from 4.92 ~ 7.97 kWh kgCOD–^1^, which exceeds the energy requirements of its treatment^8^. So, it would be exciting if a MFC-based system offers the possibility of generating enough energy for self-sufficient wastewater treatment process. In the past, neutral or positive energy balance has been demonstrated in wastewater treatment process theoretically in many reactors, such as electrochemical membrane bioreactor^9^, membrane bioelectrochemical reactor^10^, and two-stage microbial fuel cell and anaerobic fluidized bed membrane bioreactor^11^. However, there has not been actual operation of energy self-sufficient MFC-based combined system for wastewater treatment.

To achieve actual energy self-sufficient wastewater treatment process with MFC-based combined system, effective methods of boosting MFC voltage are needed. Different approaches have been used to boost MFC voltages in the past. This includes connecting multiple MFCs in series or using DC-DC converter^12^. The other method in the application is serial stacking of MFCs, although this has been proved ineffective to increase the voltage due to voltage reversal, which may lead to failure of the whole system^13^. The DC-DC converter was shown to effectively boost MFC voltage, but it also had limitations of complicated circuit and substantial energy loss in the dual boosting system. However, an alternative approach of using capacitor-based circuit proved helpful for the electricity boosting^14^. With this method, electrical energy was firstly collected in capacitors, and then dispensed intermittently with high voltage output. Capacitor were charged in parallel and discharged in series using multiple MFCs, improving voltage output with negligible energy loss^12^.

From an engineering point of view, turning wastewater treatment into an energy self-sufficient process and meeting stringent effluent discharge standard, is highly desirable but very challenging. Fortunately, prior studies using capacitor-based circuit to boost MFC voltage and MFC-based combined systems to polish the effluent have provided new opportunity. This study focuses on an innovative concept proposed for *in-situ* utilization of generated electricity to achieve an energy self-sufficient wastewater treatment process using a combined MFC and intermittently aerated biological filter (IABF) system. MFC was designed and operated for COD removal and also for electricity generation to power the pumping and aeration of IABF for a deep treatment yielding high-quality effluent. The energy balance was also analyzed in terms of energy production and energy consumption in the innovative combined system.

## Results and Discussion

### Electricity generation of the MFC

**Figure 1:**
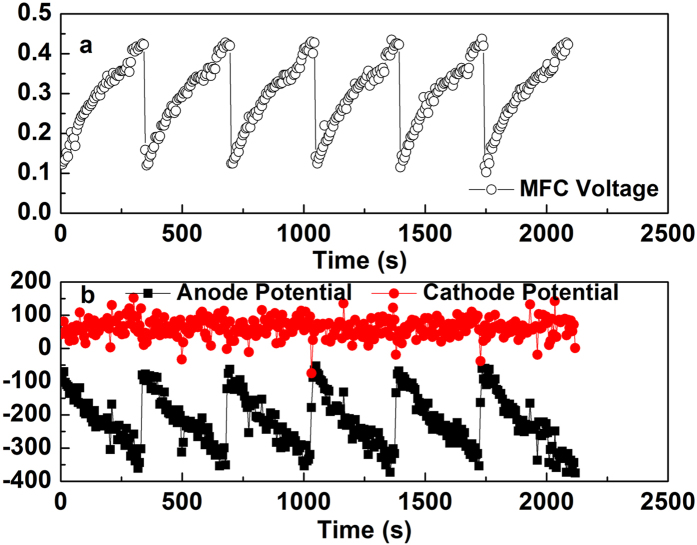
Voltage curve (**a**), anode and cathode potentials (**b**) of MFC during 6 charging and discharging cycles. Electrode potentials were reported versus the Ag/AgCl reference electrode (+197 mV vs a standard hydrogen electrode).

The electrode potentials were monitored during the whole experiment in order to investigate the performance of the system for electricity generation in capacitor-based circuit. The voltage output of MFC changed in a similar tendency in different charging and discharging cycles (Fig. 1a and Fig. S1). During the charging cycle, when MFC was connected to capacitors, the voltage of MFC rapidly decreased to 100 ± 5 mV then gradually increased to 400 ± 10 mV. When fully charged, the MFC was operated in the open circuit mode and yielded a higher voltage of 450 ± 5 mV. The anode potential dramatically decreased from −50 ± 5 mV to −350 ± 10 mV with the increase of voltage output, while the cathode potential remained nearly constant (50 ± 18 mV) (Fig. 1b). This suggests the change of voltage was attributed to the variation of anode potential. In fact, this periodic variation of anode potential indicated that the anode microorganisms displayed well-adapted performance to the drastic change of current. And the nearly constant cathode potential was related to the high catalytic activity and conductivity of cathode which was fabricated by activated carbon and polytetrafluoroethylene (PTFE)^15^.

The effect of capacitor-based circuit on increase of CE appeared to be relatively obvious. The CEs based on SCOD were 40 ± 2% under continuous flow conditions using capacitor-based circuit, and 35 ± 2% when MFCs were connected with resistances of 10 Ω. The higher CE value reflected higher electron recovery efficiency by connecting MFC reactor with capacitor than with resistor. It was speculated that with a capacitor-based circuit, a transient state (the current changed continuously) could be achieved due to repeated charging and discharging processes, compared to the steady state (the current remained relatively stable) when MFC reactor were connected with an external resistor. At steady state, current was limited by the mass transfer of exoelectrogens to the anode^14^. This mass transfer limitation could be reduced by achieving a transient state which could put the boundary layer proximity to the electrode in the dynamic changes, consequently, resulting to increased electron production^16^. In addition, higher anode potential could be achieved after discharging process (as the anode wired to the capacitor plate directly), and thus promoting exoelectrogens to produce more electrons.

### Long-term performance of MFC

**Figure 2:**
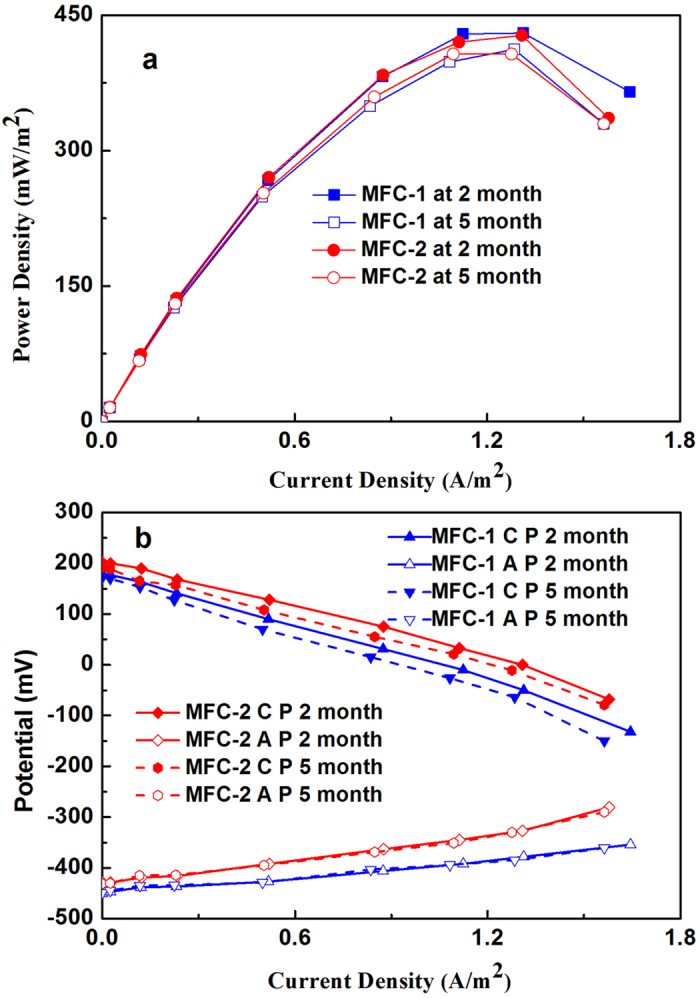
Power density, anode and cathode potentials over time of MFCs: (**a**) power density output of MFC-1, MFC-2 at 2 month and 5 month; (**b**) anode and cathode potentials of MFC-1, MFC-2 at 2 month and 5 month. The letters “A P” and “C P” in (**b**) represent anode potential and cathode potential. All electrode potentials were reported versus the Ag/AgCl reference electrode (+197 mV vs a standard hydrogen electrode).

The power density produced by MFCs changed slightly over time. The maximum power densities of each cell slightly decreased to 412 mW m^−2^ (MFC-1) and 407 mW m^−2^ (MFC-2) after 5 months compared to the data obtained after 2 months (430 mW m^−2^, MFC-1; 427 mW m^−2^, MFC-2) (Fig. 2a). Cathode potentials were slightly lower after three-month operation while anode potentials had no obvious change (Fig. 2b). The main reason for the decrease in power generation could be attributed to slight deterioration of the cathode performance. These maximum power densities of MFCs in continuous flow were lower than those previously obtained in preliminary test when the two MFCs were operated in fed-batch mode (518 mW m^−2^, MFC-1; 520 mW m^−2^, MFC-2). On the other hand, decrease in power output could be associated to dissolved oxygen penetrating to the anode chamber.

### COD removal and sludge yield of the combined system

The MFC-IABF system showed excellent treatment performance in term of effluent COD. The IABF was first inoculated with activated sludge obtained from Wenchang sewage treatment plant (Harbin, China). Some characteristics of the sludge were as follows: settled volume (SV) 24%, mix liquor suspended solids (MLSS) 2.3 g L^−1^ and sludge volume index (SVI) 104 mL g^−1^. After inoculation, the IABF was firstly operated in batch mode for 7 days without backwashing, and then turned to continuous operation fed with the effluent of MFC reactor for 20 days. During the inoculation and start-up period, the operation cycle of the aerator was fixed at 6 min including an aeration phase of 1min and a rest phase of 5min (air-liquid ratio of 12). After one month of biofilm formation and acclimation to the effluent of MFC reactor, the IABF system achieved good performance in term of effluent COD (Fig. S2).

**Figure 3:**
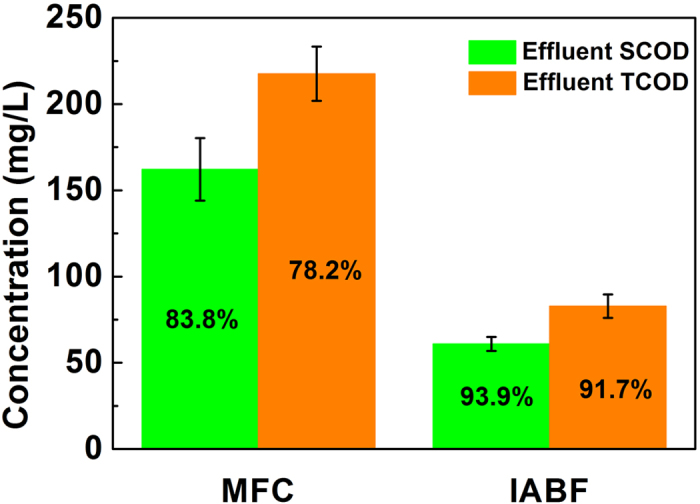
Wastewater treatment performance of combined MFC-IABF system. The value inside the figure was removal rate in terms of SCOD and TCOD (influent COD = 1000 mg/L).

A large amount of SCOD was removed (83.8%) by the MFC reactor with the effluent SCOD concentrations of 162.2 mg L^−1^. Further SCOD removal of 10.0% was achieved by the IABF and effluent SCOD of IABF realized 61 mg L^−1^ (Fig. 3). The influent TCOD decreased from 1000 mg L^−1^ to 217.6 mg L^−1^ after MFC treatment and later to 82.8 mg L^−1^ in effluent from IABF, providing an overall TCOD removal for the combined system of 91.7% (78.2% for the MFC reactor, 13.4% for the IABF) (Fig. 3). The MFC and IABF stages achieved different performance in term of substrate degradation rate (SDR). SDRs were 0.26 kg COD m^−3^ d^−1^ for the first-stage MFC, and 0.45 kg COD m^−3^ d^−1^ for the second-stage IABF, with a total SDR for the combined system of 0.28 kg COD m^−3^ d^−1^. The relatively faster substrate degradation rate reflected a higher efficiency of IABF to the entire COD removal. This wastewater treatment performance could be further enhanced through optimizing the reactor design and operating conditions including increasing volumetric ratio of MFC, increasing HRT in IABF (Table S1), and charging and discharging time optimization.

It is vital to note that conventional activated sludge process represents one of the most energy intensive operations, consuming about 3–5% of the total electrical energy load in developed countries. Meanwhile, treatment and disposal of sewage sludge pose severe challenge for wastewater treatment plants around the world^17^. A remarkable advantage of the combined MFC-IABF system to conventional activated sludge process was its high COD removal rate. COD treatment efficiency in the combined MFC-IABF achieved more than 90%, while the corresponding value for the conventional activated sludge process was relatively lower, suggesting the feasibility of the combined system as a wastewater-treatment role. Another significant advantage of the combined system is low sludge yield which is much more less than conventional activated sludge process. It is worth noting that the sludge production in this combined system was reduced by 25% with the sludge yield of 0.25 kgVSS kgCOD_removed_^−1^. This additional sludge reduction is equivalent to about 0.07 kgVSS m^−3^ d^−1^ using the combined system (detailed information and calculations in the SI), which could be highly costly in terms of treatment and disposal. Moreover, aerobic treatment process in IABF had been found to be relatively effective in terms of short detention time and high-quality effluent, compared with high-cost membranetechnology.

### Change of DO concentration in IABF

**Figure 4:**
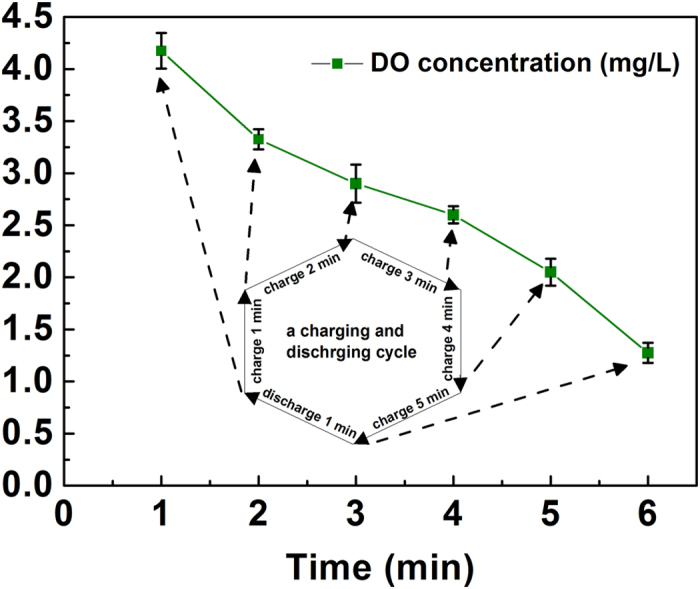
Changes of DO concentration at the bottom of IABF reactor in a charging (5 min) and discharging (1 min) cycle. The first point was measured after the capacitor-based circuit finished one-minute discharging process. The following five points were measured during five-minute charging process (one-minute interval).

Adequate aeration in IABF reactor is essential to reach the desired level of treatment, as microbe bioactivity within the reactor was markedly influenced by dissolved oxygen (DO) concentration^18^. DO concentration decreased from 4.2 mg L^−1^ to 1.3 mg L^−1^ during each changing and discharging cycle (Fig. 4), which resulted from intermittent aeration mode (aeration rate of 1000 ± 80 mL h^−1^). The DO concentration fluctuated around 2.7 mg L^−1^ compared to 2 mg L^−1^ in conventional aerobic treatment process, an indication that this could not lead to significant change in COD removal^19^,^20^. This IABF reactor operated in concert with MFC for more than 3 months without any backwashing. This is likely to be a combination of factors including small-scale reactor, use of carbon fiber as media^21^, intermittent aeration^22^, and short operating cycle. The use of MFC as the primary treatment process could contribute to this stable performance of IABF due to the removals of COD and TSS in MFC. A low sludge yield of MFC also led to the stable operation of flux through IABF without backwashing, as it is an energy-intensive process.

### Energy balance

All the volumetric energy densities were expressed on basis of normalizing to 1 m^3^ of influent. In this two-stage MFC-IABF system, energy consumption was mainly due to the pumping (feeding to the MFC reactor) and aeration in IABF system. The electrical energy produced by MFC was calculated as 0.27 kWh m^−3^ by the formula (3). Large fraction of the total energy (0.22 kWh m^−3^) was consumed for aeration in IABF. The energy consumption of pumping was 0.014 kWhm^−3^, a small fraction of the total energy consumption, which could be negligible compared to energy requirement for aeration. However, electrical energy loss occupied 13.3% (0.036 kWh m^−3^) of the whole energy consumption, and is presumed to occur when the voltage input decreased below the minimum working voltage of pump and aerator (1.5 V for aerator, and 3 V for pump). It is worth noting that energy conversion efficiencies of 4% for the pump and 3% for the aerator were assumed here in this research (detailed information and calculations in the SI). The energy balance in Table 1 was established based on the assumed *η* value.

Electrical energy was allocated to the pump or aerator depending on the change of liquid level in the head tank controlled by a float switch (FBS-70S-531A, Zetian Corporation, China). When the liquid level fell 1 mm below the height of installed switch, the capacitors were discharged through the pump. Consequently, when the liquid level rose in the head tank to the height of the installed switch, the capacitors were discharged through the aerator (Fig. S3). In the two consecutive discharging cycles (defined as stage 1 and stage 2), voltage output of capacitor-based circuit remained 5 ± 0.4 V which was applied to pump in stage 1 (lasted 1-2 s) to maintain a flow rate of 2 L d^−1^. In stage 2, electrical energy was firstly consumed by aerator before the voltage dropped below 1.5 V (lasted 10–15 s) (Fig. S4). The short time aeration was enough to obtain an aeration rate of 1000 ± 80 mL h^−1^. After that, the remaining electrical energy was released in form of heat, and the relay switched the discharging mode from powering aerator to pump because of the drop in liquid level in the head tank (see video for pumping and aeration).

The estimation of energy consumption in energy balance was complex. The main factor *η* in formulas (4) and (5) reflects energy conversion efficiency of electrical energy to pump or aeration energy, which has important impact on energy balance analysis. The efficiencies of electric motor to drive axial pump, pump impeller, and inverter are all necessary to be taken into account when the overall conversion efficiency of the pump for energy consumption calculation was assumed. So far, there are limited researches taking energy conversion efficiency *η* into consideration when estimating the energy consumption (Table 2). Typical energy consumption in these systems arises from the pumping system, which consists of three parts: influent feed, permeate, and recirculation of the electrolyte. Energy conversion efficiencies of these pumps varied between 60–100% based on theoretic analysis combined with engineering practice experience of these larger AC pumps. However, the small DC pump and aerator in this research were less efficient as compared to larger AC pump and aerator^7^ due to the combination of factors including the fluctuation of input voltage, greater pump head (0.2 m), and pipe resistance. The primary effect of inaccurately-estimated *η* might result in imprecise pumping and aeration energy, and eventually preclude a precise energy balance analysis. Taking the calculation of aeration energy consumption as an example, assuming energy conversion efficiency for the aerator of 4% compared to the value of 3%, the total electrical energy requirement for aeration could be 0.165 kWh m^−3^. This little increase of 1% in energy conversion efficiency could amount to a 20.4% decrease in the proportion of aeration consumption in total energy consumption. Therefore, estimation of energy consumption in energy balance analysis is needed for further study and in-depth analysis of the whole system.

In the present study, a combined MFC-IABF system was constructed and successfully operated in an energy self-sufficient manner at ambient temperature. The total loading of substrate degradation rate (SDR) of combined system was 0.28 kg COD m^−3^ d^−1^ (0.26 kg COD m^−3^ d^−1^ for the first-stage MFC, and 0.45 kg COD m^−3^ d^−1^ for second-stage IABF. The electrical energy produced by MFC (0.27 kWh m^−3^) was successfully used to power the pumping system (0.014 kWh m^−3^) and aeration system (0.22 kWh m^−3^). These results demonstrate that: (1) MFC technology can harvest the energy reserved in wastewater to make the wastewater treatment process with zero energy input; (2) Better wastewater treatment performance can be obtained in a MFC-based combined system, compared to that of advancing MFC technology alone. While the feasibility of the combined system had been proved, additional work will be needed to achieve more functional advantages, such as nitrification, denitrification or phosphate recovery in MFC or IABF. Also, more effective energy distribution between pump and aerator is needed to enable a sustainable operation of such a combined system.

## Methods

### Reactor construction

**Figure 5:**
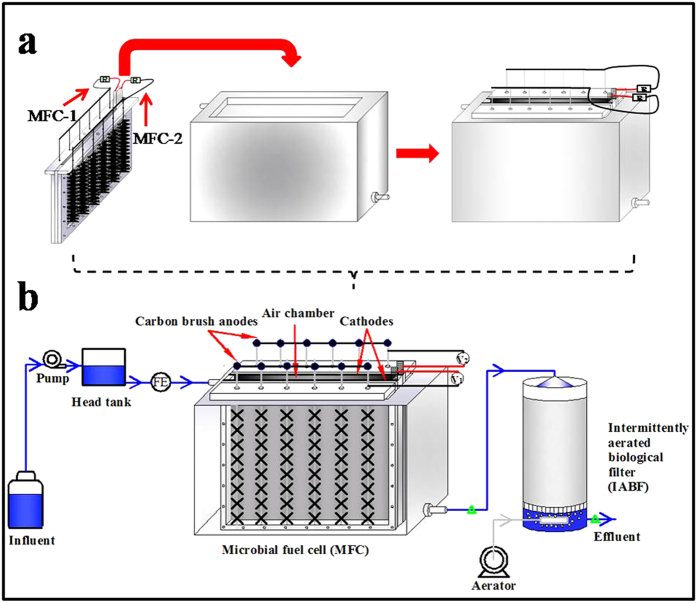
Schematic diagram of: (**a**) the MFC reactor, (**b**) the two-stage combined MFC-IABF system. The green triangles indicated the sampling points of MFC and IABF reactors for liquid quality analyses. The blue and white lines indicated the directions of liquid and air flow.

The combined MFC-IABF system consisted of one MFC and one IABF connected hydraulically (Fig. 5). The MFC contained two rows of brush anodes connected together externally with copper wire, and two pieces of rolling cathodes. The anodes were carbon fiber brushes with a titanium wire core (4 cm diameter by 20 cm length, surface area of 2.41 m^2^) that were heat treated at 450 °C for 30 min before use. Cathodes (30 × 20 cm, cathode surface area = 600 cm^2^) were made by “rolling-press” method using activated carbon and PTFE^23^. This MFC was placed in a rectangular box (40 × 10 × 20 cm), with an effective volume of 6 L (Fig. S5 and Fig. 5a). The IABF reactor (10 cm inside diameter, 20 cm in height) was made of Plexiglas and filled with carbon brushes which were used as filter material, with an effective volume of 600 mL. An air diffuser (10 cm outside diameter, Xiangsu Corporation, China) was located at the bottom of the reactor to inject air. In order to support the filter media and ensure a well-distributed air supply, a porous gas distribution plate (10 cm outside diameter, pore size 3 mm) was placed up the air diffuser. Effluent of MFC flowed downward through IABF reactor, and the aerator (SC3301PM, Lichang Corporation, China) (Table S2 and Fig. S6) worked intermittently to inject air upward.

### Operation conditions

The MFC reactor was inoculated with the effluent of an existing MFC operated at ambient temperature. During the first two months, the two independent MFCs (MFC-1 and MFC-2 in Fig. 5a) were firstly acclimated at external resistance of 500 Ω, and then the external resistance consistently switched to 10 Ω to maximize power generation. The medium contained glucose (1 g L^−1^) in a 50 mM phosphate buffer solution (PBS) and also containing trace minerals and vitamins as previously described^24^.

Two MFCs were changed to a parallel connection to charge the capacitors when combined with IABF. A DC pump (Mini260, Aoqi Corporation, China) (Table S2 and Fig. S6) was used to transport the influent from a container into a head tank placed 20 cm above the container, and then it flowed from the head tank through a liquid flow meter (LZB-2, Flowtech Corporation, China) into the upper-left of MFC reactor due to the hydraulic pressure. Then, the effluent from the lower-right of the MFC reactor flowed into the top of the downstream IABF reactor, due to the hydraulic pressure (Fig. 5b). The effluent samples of MFC reactor and IABF reactor for COD analysis were taken every 10 days during the three-month operation.

### External circuit design

A capacitor-based circuit was used to harvest electrical energy from the MFC reactor (Fig. S7). The circuit was made up of capacitors (3.3 F, Panasonic Corporation, Japan) and relays controlled by programmable microcontroller (XD-J16H, Xunda Corporation, China). The MFCs charged the capacitors (5 min, optimal process in Fig. S8) in parallel and charged capacitors connected in series to power the pump and aerator (1 min).

### Measurement, analyses and calculation

Total COD (TCOD) and soluble COD (SCOD) were measured using standard methods. The samples for SCOD measurement were firstly filtered through a 0.45 μm pore diameter syringe filters. The dissolved oxygen concentration was measured by DO meter (in Lab Oxi730, WTW, Germany).

The voltages across the MFC were recorded using a data acquisition system PISO-813 (32 Channel ICP DAS Co., Ltd.). Polarization and power curves were obtained by changing the external resistance from open circuit to 2.7 Ω under continuous flow condition.

The capacitors were charged and discharged cyclically from a discharging potential (*V*_*d*_) to a charging potential (*V*_*c*_).

The quality of charge (*Q*) harvested in a charging cycle can be calculated with the following formula (1).


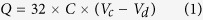


Where *C* represents the capacitance (3.3 F) and 32 represents the number of capacitors parallel connected.

The coulombic efficiency (CE) was calculated based on SCOD removal in MFC by formula (2)^25^.


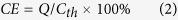


Where *C*_*th*_ represents total charge available based on substrate consumption.

The energy (*W*_*c*_) harvested from the MFC in a single charging cycle was calculated using formula (3)^26^.


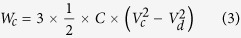


The electrical energy consumption of pump was estimated as formula (4)^27^.


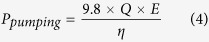


Where 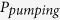 represents electrical energy consumption of the pump (kW), *Q* is influent flow rate (m^3^ s^−1^), *E* is the hydraulic pressure head (m), *η* is the energy conversion efficiency of the pump. In this study, *Q* was 2.3 × 10^−8^ m^3^ s^−1^ (1.39 mL min^−1^) for influent flow rate. The hydraulic pressure head was 0.2 m.

The electrical energy consumption of aerator was estimated as formula (5)^28^.


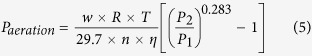


Where 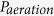 represents electrical energy consumption of the aerator (kW), *w* in weight of air flow (kg s^−1^), _*R*_ = gas constant, 8.314 kJ kmol^−1^ K^−1^, *T* = absolute inlet air temp (298 K), *n* = constant for air or 0.283, *η* = efficiency of the aerator, *P*_2_ and *P*_1_ are absolute outlet and inlet pressure (atm), respectively.

## Additional Information

**How to cite this article**: Dong, Y. *et al.* A combined system of microbial fuel cell and intermittently aerated biological filter for energy self-sufficient wastewater treatment. *Sci. Rep.* **5**, 18070; doi: 10.1038/srep18070 (2015).

## Supplementary Material

Supplementary Information

Supplementary Video

## Figures and Tables

**Table 1:** Energy production and consumption in combined MFC-IABF system.

Feeding Energy	*Q* (m^3^s^−1^)		*E*(m)		*η*^a^			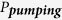 (kW)	Energy consumption (kWh m^−3^)
	2.3 (10^−8^)		0.2		4%			1.1 (10^−6^)	1.4 (10^−2^)
Aeration Energy	*w* (kg s^−1^)^b^	*R* (kJ kmol^−1^ K^−1^)	*T* (K)	*n*	*P*_1_ (atm)	*P*_2_ (atm)^c^	*η*^d^	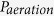 (kW)	Energy consumption (kWh m^−3^)
	3.3 (10^−7^)	8.314	298	0.283	1	1.02	3%	1.8 (10^−5^)	0.22
Produced energy(kWhm^−3^)									0.27

^a^Assume energy efficiency of 4% in conversion of electrical energy to pump energy.

^b^Standard air has a specific weight of 1.2 kg m^−3^ (298 K, 1 atm, and a relative humidity of 36%).

^c^*P*_2_ was calculated depending on the depth of IABF reactor, without considering other pressure loss.

^d^Assume energy efficiency for aerator of 3%.

**Table 2:** Comparison of pump efficiency *η* in different reported systems.

System	Energy consumption	Energy efficiency (*η*)	Reference
EMBR	Pumping	64.6%	9
MBER	Pumping	100%	10
MFC –AFMBR	Pumping	65%	11
AFMBR	Pumping	64.6%	27
MFC-IABF	Pumping and aeration	4% and 3%	This research
