# Supplementary material for: A combined system of microbial fuel cell and intermittently aerated biological filter for energy self-sufficient wastewater treatment
Source: Sci Rep. 2015 Dec 15;5:18070. doi: 10.1038/srep18070 (PMC4678297; doi:10.1038/srep18070)
Supplement: Supplementary Information [file srep18070-s1.pdf]

Supplementary Information

A combined system of microbial fuel cell and intermittently aerated biological filter for energy self-sufficient wastewater treatment

Yue Dong[1], Yujie Feng[1,*], Youpeng Qu[2], Yue Du[1], Xiangtong Zhou[1] & Jia Liu[1]

[1]State Key Laboratory of Urban Water Resource and Environment, Harbin Institute of Technology. No 73 Huanghe Road, Nangang District, Harbin 150090, China
[2]School of Life Science and Technology, Harbin Institute of Technology. No. 2 Yikuang Street, Nangang District, Harbin 150080, China

*Corresponding Author:
E-mail: yujief@hit.edu.cn; phone: (+86)451-86287017;
Fax: (+86) 451-86287017

Table S1. The effluent COD at increased HRTs in IABF

HRT (h)	8.5	9	9.5
Effluent TCOD (mg/L)	59 ± 10	49 ± 8	40 ± 5
Effluent SCOD (mg/L)	45 ± 3	37 ± 4	31 ± 3

Table S2. Specifications of DC pump and aerator in this paper

**Pump specifications**		
Operating voltage	V (V)	3-6 V
Operating current	I (A)	0.09-0.2 A
Operating power	p (W)	0.27-1.2 W
Maximum flowrate	Q_{max} (L/min)	2 L/min (pump head: 0.2 m)
Maximum pump head	H_{max} (m)	0.5 m
**Aerator specifications**		
Operating voltage	V (V)	1.5-6 V
Operating current	I (A)	0.125-0.265 A
Operating power	p (W)	0.188-1.59 W
Maximum aeration flowrate	Q_{max} (L/min)	0.7 L/min (aeration depth : 0.2 m)
Maximum aeration depth	D_{max} (m)	0.8 m

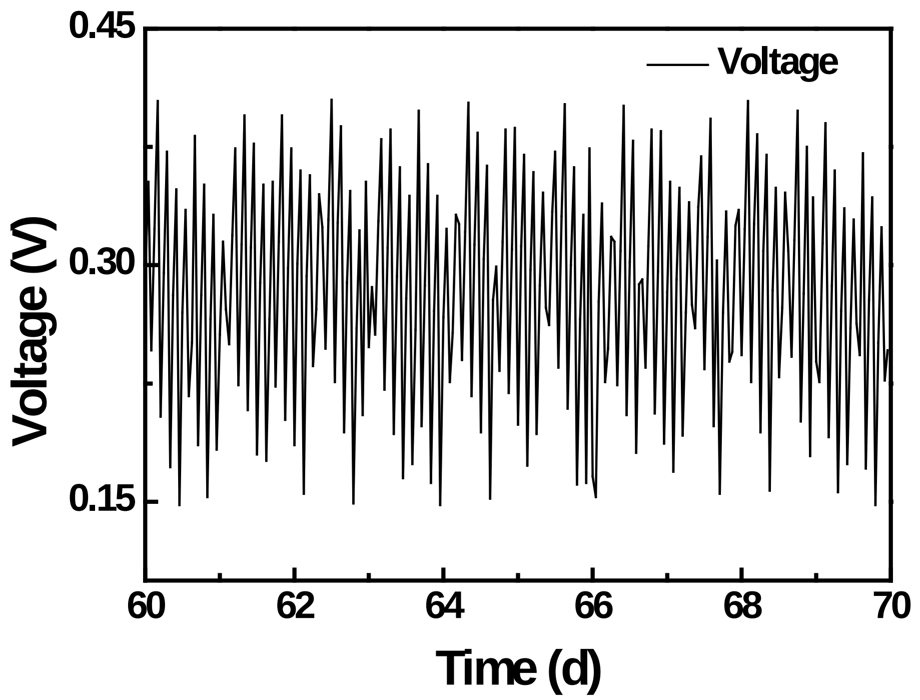

Figure S1. Part of the voltage data that was produced by MFCs during the charging and discharging cycles during the three month operation of MFC-IABF system (from 60 to 70 days).

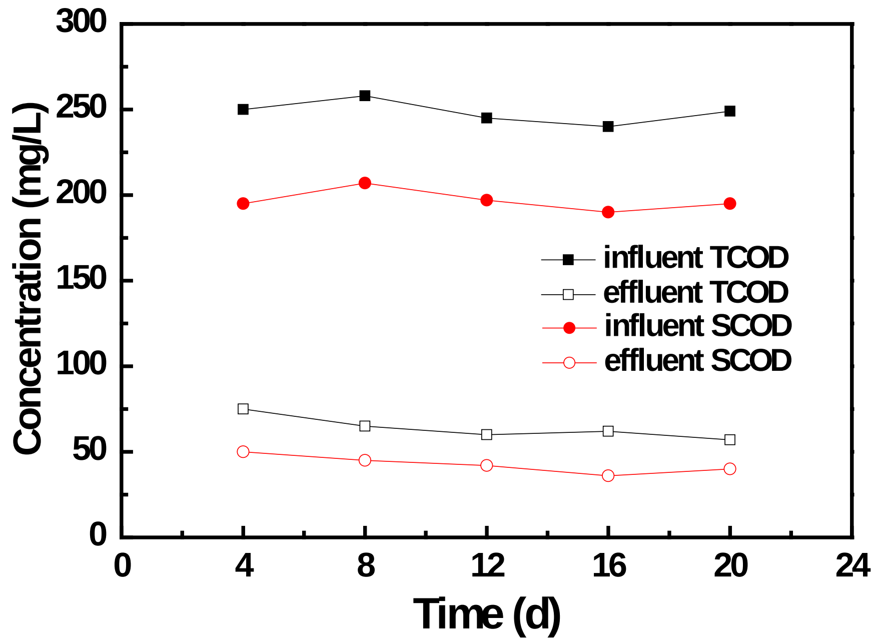

Figure S2. COD removal in the strat-up period (20 d) of the IABF reactor.

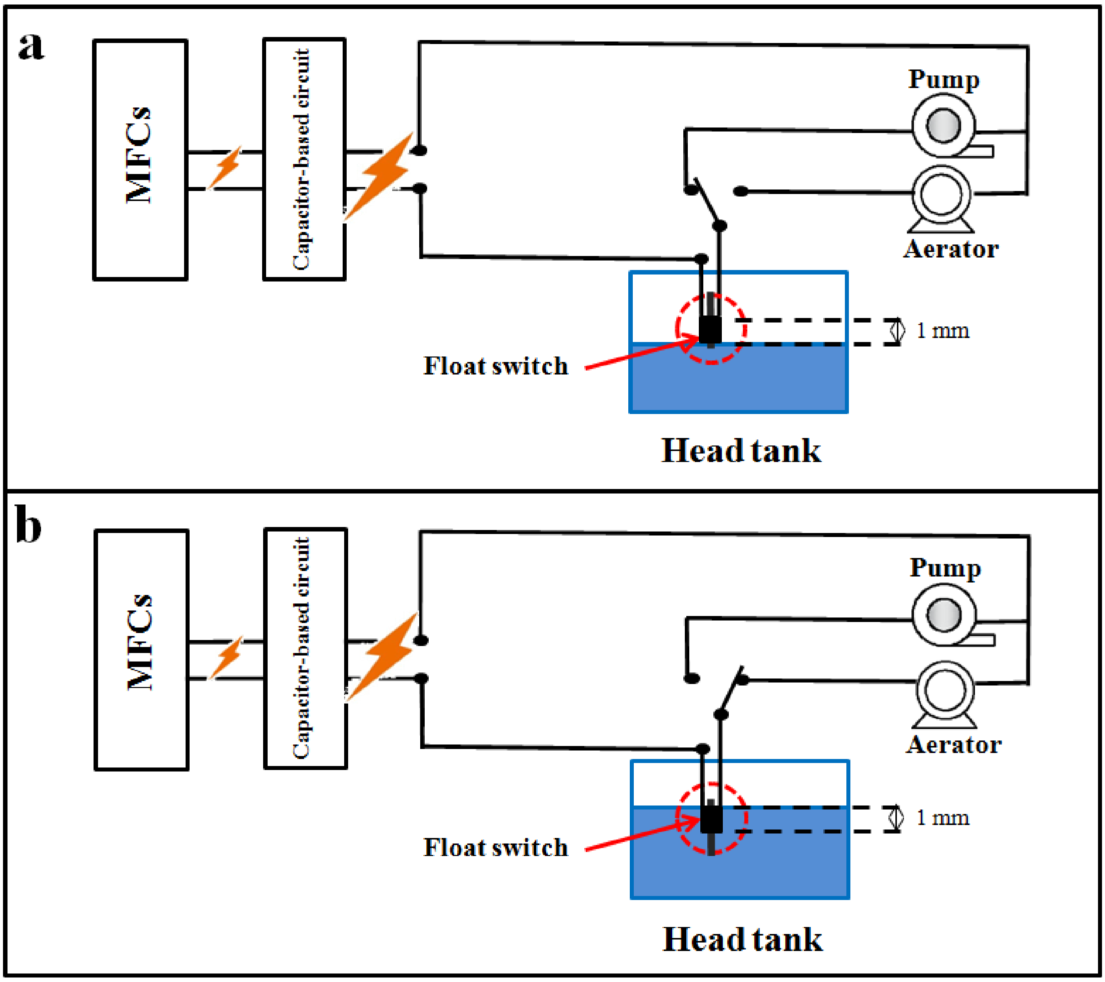

Figure S3. A float switch controlled liquid levels within 1 mm in the head tank, allocated electrical energy solely to the pump or the aerator. When the liquid level fell 1 mm below the height at which the switch installed, the capacitors were discharged through the pump (a). When the liquid level rose in the head tank to the height at which the switch installed, the capacitors were discharged through the aerator (b).

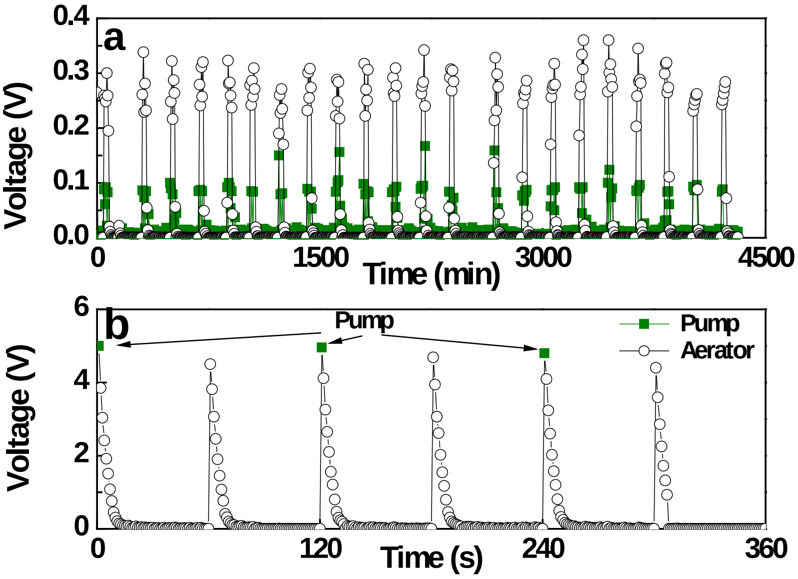

Figure S4. The macroscopic changes of operating voltage across pump and aerator over 3 days (a). The microcosmic changes during 6 consecutive discharging cycles (b).

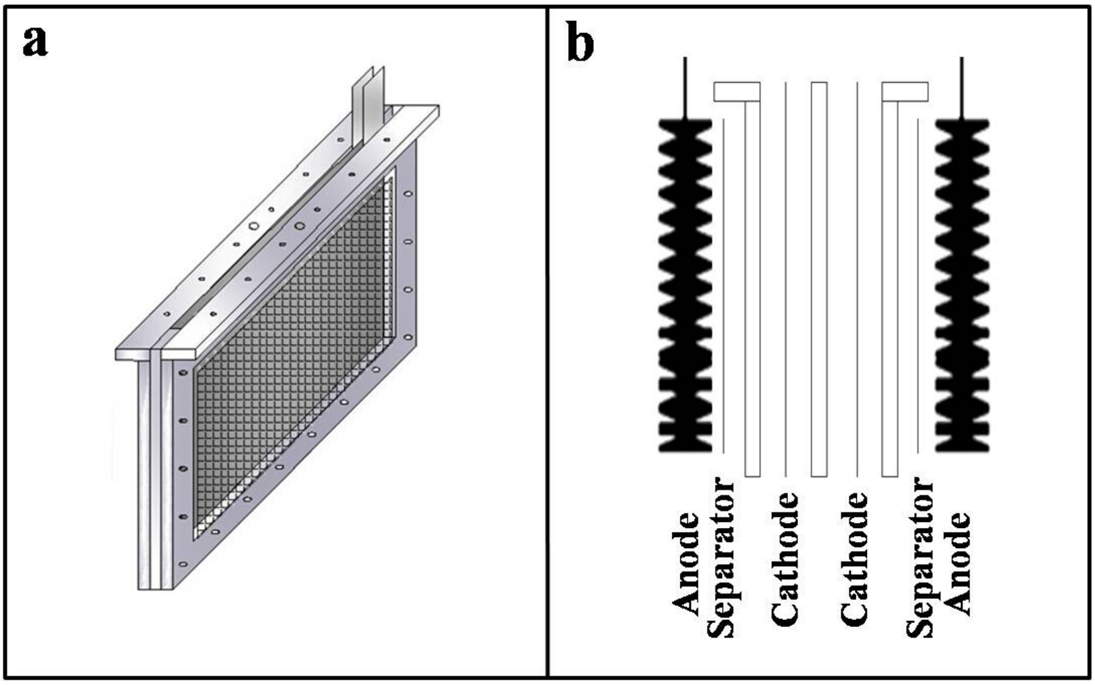

Figure S5. Schematic diagram of MFCs assembled: (a) front view of the MFCs without anode, (b) side view of the MFCs with anode.

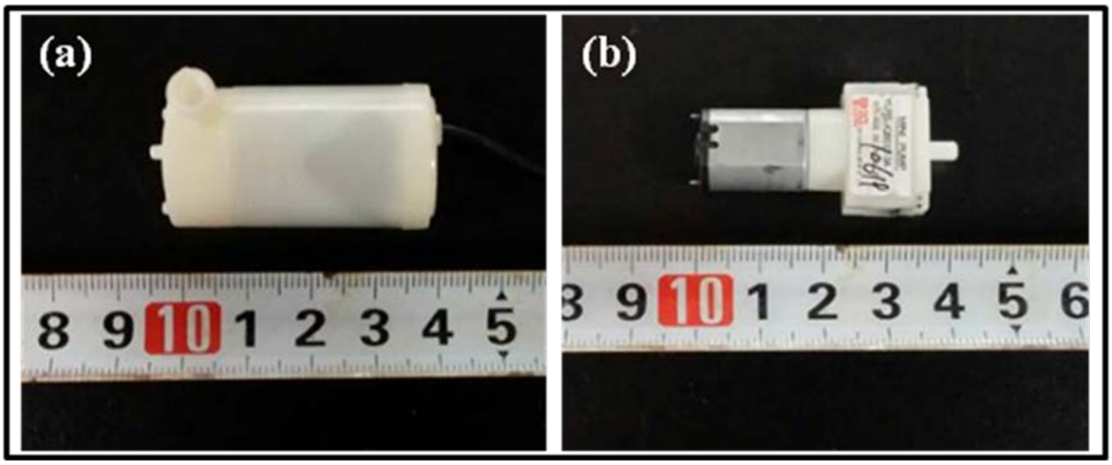

Figure S6. Photos of pump (a) and aerator (b).

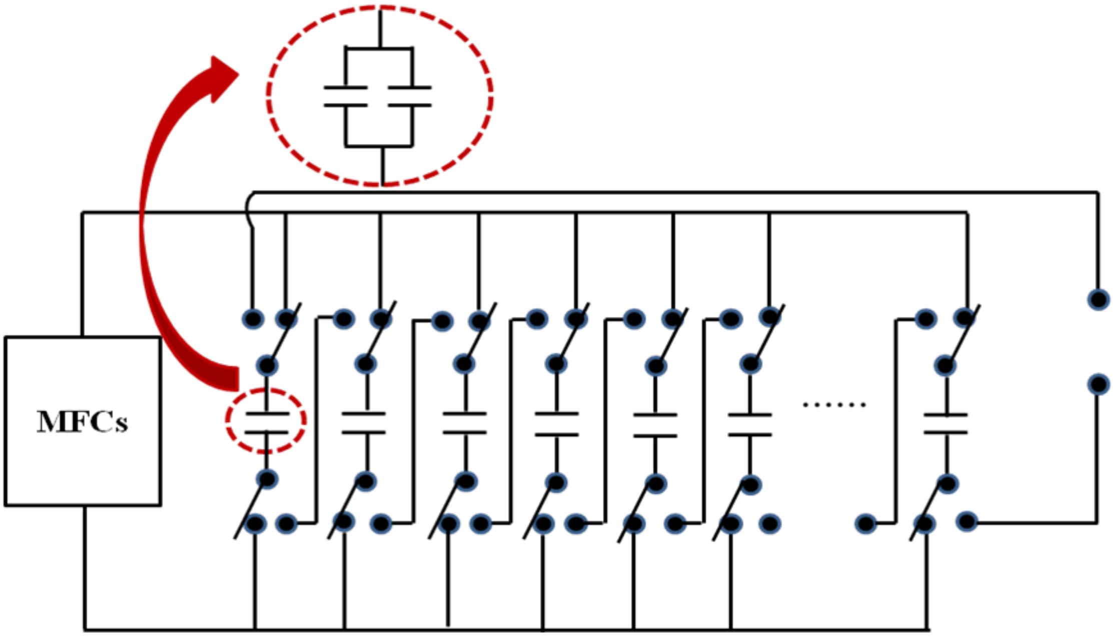

Figure S7. Capacitors (32 capacitors with every 2 capacitors connected in parallel, 16 groups, 3.3 F/2.5V) were charged by the two MFCs connected in parallel and discharged in series to the pump by the control of relays (a charging and discharging cycle was fixed at 6 min including a charging phase of 5 min and a discharging phase of 1 min.

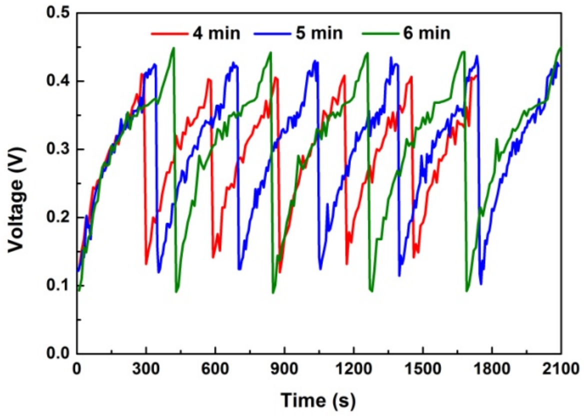

Figure S8. The changes of voltage across the MFC under the charged time of 4 min. 5 min, and 6 min.

Estimation of sludge yield in the combined MFC-IABF system

MFC and IABF represent two different treatment processes, thus have different sludge yields. So, we should estimate the sludge yield of the two stages, separately.

For first-stage MFC, the sludge yield varied between 0.07 – 0.22 kgVSS $kgCOD_{removed}^{-1}$ fed with glucose[1]. For the consensus that aerobic removal of COD could be sustained by oxygen diffusion through the cathode, and inevitably some of the oxygen will be used for biomass production. We selected maximum value of 0.22 kgVSS $kgCOD_{removed}^{-1}$ as the parameter for the estimation.

For second-stage IABF, it was reported that the sludge yield was 0.41 kgVSS $kgCOD_{removed}^{-1}$ with domestic wastewater[2]. Little information on sludge yield with glucose as substrate is available. However, as a typical domestic wastewater has a COD concentration of 500 mg L^{-1}, which was higher than the influent concentration to the IABF of 217.6 mg L^{-1}, it is reasonable to assume the sludge yield will lower than that with domestic wastewater[3]. From the point of precise analysis, we still use 0.41 kgVSS $kgCOD_{removed}^{-1}$ as the parameter for calculation.

Thus, the sludge yield of this combined MFC-IABF system is:

[(0.22 kgVSS kgCOD $_{removed}^{-1}$) × (1000-217.6) × 10^{-6} kg L^{-1} + (0.41 kgVSS kgCOD $_{removed}^{-1}$) × (217.6-82.8) × 10^{-6} kg L^{-1}] / [(1000-82.8) × 10^{-6} kg L^{-1}] = 0.25 kgVSS kgCOD $_{removed}^{-1}$

A typical sludge yield with conventional activated sludge process is 0.5 kgVSS kgCOD $_{removed}^{-1}$, so the sludge yield with the combined system is only a half of it[4].

Taking the total substrate degradation rate (SDR) of combined system of 0.28 kg COD m^{-3} d^{-1} into consideration, the reduction of sludge production was 0.07 kgVSS m^{-3} d^{-1}, which represented a high cost for treatment and disposal it.

Estimation of energy conversion efficiencies (η) for the pump and aerator

In order to calculate the energy consumption of the pump and aerator, the energy conversion efficiencies were evaluated.

The operating voltage of the pump was 3 – 6 V, with the operating current varied between 0.09 – 0.2 A (the corresponding values for the aerator were 1.5 -6 V and 0.125 – 0.625 A). And the energy conversion efficiencies of the pump and aerator all decreased non-linearly with the increase of voltage from 3 V to 6 V for the pump and 1.5 V to 6V for the aerator.

In this research, the maximum voltage output of the capacitor-based circuit was around 5 V, and then it gradually decayed to the minimum operating voltage of 3 V for pump or 1.5 V for aerator, which was in the range of the operating voltage of the pump or aerator. So, it is reasonable to assume that the energy conversion efficiencies

of these devices will be between those of maximum efficiencies and minimum efficiencies. Thus, the average values were used for the estimation of energy conversion efficiencies of the pump and aerator.

The maximum efficiency of the pump was measured as 5.2% at applied voltage of 3 V and hydraulic pressure head of 0.2 m. The minimum efficiency was measured as 2.9% at applied voltage of 6 V and hydraulic pressure head of 0.2 m. Therefore, the energy conversion efficiency of pump was estimated as:

$$\eta_{pumping} = (2.9\ \% + 5.2\ \%) / 2 = 4\ \%$$

The maximum efficiency of the aerator was measured as 3.7 % at applied voltage of 3 V and aeration depth of 0.2 m. The minimum efficiency was measured as 2.2 % at applied voltage of 6 V and aeration depth of 0.2 m. Therefore, the energy conversion efficiency of pump was estimated as:

$$\eta_{aeration} = (2.2\ \% + 3.7\ \%) / 2 = 3\ \%$$

Video supplementary material

Video for pumping and aeration.

References

1. Rabaey, K., Lissens, G., Siciliano, S. D. & Verstraete, W. A microbial fuel cell capable of converting glucose to electricity at high rate and efficiency. *Biotechnol. Lett.* **25**, 1531-1535 (2003).
2. Mendoza-Espinosa, L. & Stephenson, T. A review of biological aerated filters (BAFs) for wastewater treatment. *Environ. Eng. Sci.* **16**, 201-216 (1999).
3. McCarty, P. L., Bae, J. & Kim, J. Domestic Wastewater Treatment as a Net Energy Producer–Can This be Achieved? *Environ. Sci. Technol.* **45**, 7100-7106 (2011).
4. Su, X., Tian, Y., Sun, Z., Lu, Y. & Li, Z. Performance of a combined system of

microbial fuel cell and membrane bioreactor: wastewater treatment, sludge reduction, energy recovery and membrane fouling. *Biosens. Bioelectron.* **49**, 92-98 (2013).
